# Natural Killer T (NKT) Cells in Autoimmune Hepatitis: Current Evidence from Basic and Clinical Research.

**DOI:** 10.3390/cells12242854

**Published:** 2023-12-18

**Authors:** Dimitri Poddighe, Tilektes Maulenkul, Gulsamal Zhubanova, Lyudmila Akhmaldtinova, Kuanysh Dossybayeva

**Affiliations:** 1 School of Medicine, Nazarbayev University, Kerei-Zhanibek Str. 5/1, Astana 010000, Kazakhstan; 2 Clinical Academic Department of Pediatrics, National Research Center for Maternal and Child Health, University Medical Center, Astana 010000, Kazakhstan

**Keywords:** natural killer T cells, NKT cells, autoimmune hepatitis, children, adults, murine models, Concanavalin A, α-Galactosyl-ceramide, liver inflammation

## Abstract

Natural killer T (NKT) cells are unconventional T cells that are activated by glycolipid antigens. They can produce a variety of inflammatory and regulatory cytokines and, therefore, modulate multiple aspects of the immune response in different pathological settings, including autoimmunity. NKT cells have also been implicated in the immunopathogenesis of autoimmune hepatitis, and in this review we summarize and analyze the main studies investigating the involvement and/or homeostasis of NKT cells in this disease. In detail, the evidence from both basic and clinical research has been specifically analyzed. Even though the experimental murine models supported a relevant role of NKT cells in immune-mediated hepatic injury, very few studies specifically investigated NKT cell homeostasis in patients with autoimmune hepatitis; however, these initial studies reported some alterations of NKT cells in these patients, which may also correlate with the disease activity to some extent. Further clinical studies are needed to investigate the potential role and use of NKT cell analysis as a disease marker of clinical relevance, and to better understand the precise cellular and molecular mechanisms by which NKT cells contribute to the pathogenesis of autoimmune hepatitis.

## 1. Introduction

Natural killer T (NKT) cells are lymphocytes expressing both T and NK cell markers, which specifically recognize glycolipids presented by the monomorphic MHC-like molecule CD1d [1,2]. Therefore, NKT cells are included in the group of “unconventional” T cells due to their peculiar immunophenotypic and immuno-functional characteristics, which put them between innate and adaptive immunity [1,2].

Autoimmune hepatitis (AIH) is a chronic inflammatory disease caused by a loss of tolerance to liver self-antigens, leading to the appearance of several autoantibodies. They are used as diagnostic markers to recognize this liver disease. AIH affects both children and adults, and has been described in all ethnicities [3].

A number of reviews discussed the immunological mechanisms implicated in the pathogenesis of autoimmune liver disease, including the potential contribution of NKT cells. Most of these articles are narrative descriptions composed by merging different types of experimental evidence [4,5,6].

In this review, we aim to summarize the current evidence directly supporting the potential implication of NKT cells in AIH by clearly differentiating and distinguishing the information from the most commonly used experimental model to study autoimmune liver disease (namely mice exposed to Concanavalin A), and that emerging from clinical (human) studies, in order to specifically highlight the findings related to NKT cell homeostasis and function according to the specific research resource and setting.

### 1.1. Natutal Killer T Cells

The NKT cell population in mice was first identified in 1987, when different research groups reported the existence of a T cell subset characterized by intermediate TCR expression and “preferential” use of a TCRβ chain including the variable (V) β8 domain [7,8,9]. Eventually, NK markers were identified on these unusual T cells, which were defined as CD3^+^TCRαβ^+^NK1.1^+^ T cells in mice [10,11]. In the following years, similar cells were also identified in humans (which were positive for CD161-positive, corresponding to NK1.1 in mice) [12,13].

The term “NKT cells” to define a distinct lymphocyte population with the aforementioned immunophenotypic characteristics was first used in 1995. These cells were rapidly recognized to have modulatory properties on the immune response through the production of several cytokines [14]. However, the main step in the understanding of the immunobiology of these cells was the observation that they were reactive to the MHC class-I-like molecule CD1d, thanks to the expression of an invariant TCR α-chain consisting of Vα14-Jα281 (also known as Jα18) in addition to the presence of a TCR β-chain including Vβ8.2 domain [14,15,16].

Eventually, two broad classes of NKT cells have been defined both in mice and humans. Type I NKT cells (also called “invariant” NKT—iNKT—cells) are characterized by the specific recognition of the lipid antigen α-galactosyl-ceramide (αGalCer) through the expression of a specific CD1d-restricted semi-invariant αβTCR, which in humans is composed by the invariant Vα24–Jα18 α-chain and a β-chain including the Vβ11 domain. In mice, this invariant αβTCR includes the Vα14–Jα18 α-chain and a β-chain with a limited set of variable domains (Vβ8, Vβ7, and Vβ2) [14,17]. Type II NKT cells can express a different, but limited, TCR repertoire, which allows them to recognize both glycolipids and phospholipids presented by CD1d overall. However, their precise antigen specificities are not well or completely defined yet. Because of this knowledge gap, type II NKT cells are more difficult to identify and investigate [1,14,17]. Indeed, type I NKT cells can be reliably identified by using CD1d/αGalCer tetramers, through the monoclonal antibody clone 6B11 (targeting the conserved CDR3 region of the Vα24-Jα18 TCR), or by reactivity to synthetic αGalCer (loaded on a dimer formed by CD1d:Ig and β2-microglobulin) [1,18,19]. Conversely, type II NKT cells lack any reliable positive markers. Therefore, their identification is challenging and could be based on the exclusion of the TCR-Vα24^+^ type I NKT cells (and, possibly, TCR-Vα7.2^+^ mucosal-associated invariant T cells as well) in the CD3^+^CD56^+^CD161^+^TCRγδ^−^ T cell population, for instance [20]. Type I NKT cells are relatively abundant in mice, representing between 1% and 3% of T cells in most tissues. However, these cells are less frequent in humans, where they account for <1% of T cells in the blood and liver. Indeed, humans have a greater proportion of type II NKT cells compared to mice [17].

Information about the ontogeny of NKT cells is mainly available for type I and comes from murine studies. The thymic NKT cell development pathway includes several stages, numbered from 0 to 3, characterized by a different expression of CD24, CD44, and NK1.1 immunophenotypic markers. Then, mature type I NKT cells are further categorized according to their cytokine profile and transcription factors as NKT1 (expressing high levels of IFN-γ), NKT2 (producing IL-4), and NKT17 (characterized by the predominant production of IL-17). With respect to the aforementioned phenotypic markers, NKT1 cells are mainly NK1.1^+^CD44^hi^ (corresponding to stage 3), whereas NKT2 and NKT17 cells are both NK1.1^−^ (which characterize stages 1 or 2) [17,21].

Therefore, NKT cells can produce large amounts of IFN-γ, but they are also able to release IL-4 and other Th2 cytokines (including IL-13). The different profiles of cytokine production may depend on several factors, also related to the specific antigenic stimulation and exposure. In detail, the route of administration of the lipid antigen (e.g., systemic vs. mucosal) could modify the “Th1/Th2” cytokine polarization of NKT cells. Moreover, the specific composition of lipidic antigens (with variable length and/or saturation level of their chains) may also elicit different cytokine production from NKT cells [22,23,24,25]. Through their restricted innate-like antigenic recognition patterns and their variable cytokine release, NKT cells could modulate the adaptive immune response and its polarization during the early phase of several types of infections, since the array of glycolipids and phospholipids are variably expressed by several viruses, bacteria, and other microbial agents. However, their immunological relevance goes beyond the immune response to infections. In fact, NKT cells have been implicated in several and different pathological settings, including malignancies, autoimmunity, allergy, atherosclerosis, and transplants [26].

### 1.2. Autoimmune Hepatitis

Autoimmune hepatitis (AIH) is a chronic inflammatory disease defined by the evidence of autoantibody production against liver antigens (“seropositive”) or, in its absence (“seronegative”), by consistent histological characteristics without any other explanatory cause, such as concomitant metabolic, infectious, or oncological disease. Indeed, hyper-gammaglobulinemia, a specific autoantibody profile, consistent liver histology, and the absence of viral hepatitis markers (and for other hepatic disorders) are the main aspects that support the diagnosis of AIH [27,28,29].

Seropositive AIH represents the majority of cases, and it is classified into two main subtypes, according to the autoantibody detected in these patients: AIH type 1 (AIH-1) is diagnosed when patients are positive for the presence of smooth muscle antibodies (anti-SMA) and/or antinuclear antibodies (ANA). Type 2 (AIH-2) is diagnosed in patients showing positive results for liver kidney microsomal antibody type 1 (anti-LKM-1) and/or anti-liver cytosol type 1 (anti-LC1). Recently, some researchers suggested creating a third and separate category based on the positivity of soluble liver antigen or liver pancreas antigen, which is usually associated with AIH-1 [28]. AIH can arise in both pediatric and adult patients; however, 40% of AIH-1 and 80% of AIH-2 patients are respectively diagnosed in pediatric patients (age < 18 years) [28,30]. 

In general, AIH is attributed to a multifactorial etiopathogenesis; therefore, in each patient, genetic predisposition and exposure to potential triggering factors are implicated. The genetic factors are in part related to HLA, which is mainly linked to specific HLA-DRB1 polymorphisms for both AIH types. However, different associations with HLA-DRB1 allelic variants have been variably described according to AIH type, age, and geographical factors. With respect to environmental factors, viruses and (intestinal) microbiota have been mainly implicated in AIH etiology [3]. In this regard, molecular mimicry mechanisms have been suggested to be implicated in the immunopathogenesis of AIH. For instance, the liver enzyme cytochrome P450-2D6 (CYP2D6), which is targeted by the anti-LKM1 antibody in type 2 AIH patients, displays a significant level of homology with several viral (HCV, EBV, CMV, and others) antigens [31,32,33]. One experimental study observed a reduced diversity and total load of intestinal microbiota in a transgenic mouse model developing immune-mediated hepatitis with production of ANA and anti-LKM1/anti-LC1 antibodies [34]. Moreover, one human study also suggested the presence of gut dysbiosis with a reduced presence of anaerobes and a decreased *Bifidobacteria* spp./*Escherichia coli* ratio in patients with AIH compared to controls [35].

## 2. NKT Cells in Autoimmune Hepatitis: Evidence from Basic Research

NKT cells are mainly tissue-resident lymphocytes: as already mentioned, in mice they can represent 1–3% of T cells in most tissues, but can reach up to 50% in the liver [17,36]. 

Concanavalin A (ConA)-induced hepatitis in mice represents the main experimental model used to study AIH immunopathogenesis. ConA is a plant lectin with inflammatory and immunomodulatory properties that is able to activate T cells and macrophages. Notably, it produces liver injury with pathological changes that are in part comparable to those observed in human AIH, even though the precise mechanisms of ConA-induced hepatitis have yet to be unveiled [37]. However, it seems that ConA can bind mannose receptors in order to activate macrophages and neutrophils (which can produce superoxide anions and inflammatory cytokines), but it may also directly interact with TCRs and, thus, activate T cells. Moreover, ConA could also promote the formation of microthrombi in the hepatic sinusoid by interfering with the coagulation factors [38]. 

Despite the general limitations of murine models and other limitations specific to ConA-induced hepatitis for the investigation of AIH, this murine hepatitis represents an example of liver injury characterized by strong cell-mediated immune system activation in this organ along with concomitant and abundant production of (inflammatory) cytokines. Although it does not exactly reproduce the immuno-pathogenesis of human AIH, it allowed researchers to investigate the role of conventional T helper and cytotoxic lymphocytes in this pathological context [39,40]. However, unconventional T cells and, in detail, NKT cells have also emerged as an important lymphocyte population in the liver, where they are particularly abundant, despite their rarity in the peripheral blood [17]. In this model, NKT cells are particularly abundant in microvascular spaces and can become activated by the recognition of lipid and other antigens (also through the expression of toll-like receptors). Therefore, they are supposed to have a prominent role in the physiologic immune tolerance at the hepatic level and, conversely, in inflammatory responses in the context of autoimmune liver diseases [41]. 

In Table 1, we summarized all the main studies where NKT cells were specifically investigated in murine ConA-induced hepatitis as the main experimental model of AIH [42,43,44,45,46,47,48,49,50,51,52,53,54,55,56,57,58,59].

The investigation of ConA-induced hepatitis in both wild-type and transgenic mice indicated a prominent role of NKT cells in the development of the immunological and inflammatory responses leading to this disease. After the initial observation by Toyabe et al. suggesting the requirement of NKT cells for the development of ConA-induced hepatitis [42], the experiments by Kaneko et al. reported that NKT cells could be responsible for this hepatitis in RAG-deficient mice [43]; moreover, Takeda et al. showed that CD1d-deficient mice developed a markedly reduced disease compared to the wild-type mice [44]. Other studies showed that, in this pathological context, experimental conditions inducing an amplification or mitigation of the liver injury were respectively associated with an increase or reduction of NKT cells (or their function) [55,57]. Indeed, Tabet et al. observed that chlordecone (an organochlorine that accumulates in the liver and can potentiate the hepatoxicity of other substances) could amplify the ConA-related hepatic inflammation and also noticed that only NKT cells (among all the immune cells) were numerically and significantly increased in the inflammatory infiltrate of mice exposed to chlordecone compared to unexposed mice [54]. Conversely, Gao et al. showed that diammonium glycyrrhizinate was able to mitigate the liver inflammation in ConA-treated mice, and this improvement was also associated with a reduction of NKT cell proliferation and number in the liver [57].

Many studies applied the ConA-induced hepatitis model to knock-out mice for a number of molecules implicated in different intracellular signaling pathways in order to investigate their specific contribution to NKT cell functioning. In this regard, several studies reported a correlation between the changes in NKT cell number and/or activation and the expression and severity of liver injury overall [47,50,51,53,58]. 

Other research has highlighted the role of inflammatory and/or immunomodulatory interleukins production by NKT cells (especially IFN-γ, IL-4, TNF-α, and IL-10) in the pathogenesis of ConA-induced hepatitis [45,46,48,52,55]. Among them, Li et al. also suggested an important inhibitory role of IL-15 on the NKT cell-related production of several inflammatory cytokines in ConA-induced hepatitis. In detail, they subcutaneously administered IL-15 as a pre-treatment to ConA-exposed mice and observed a dose-dependent protective effect against liver injury, which was associated with a reduction of IL-4, IL-5, and TNF-α serum levels. Notably, they showed that NKT cells were the main source of these cytokines in the inflamed liver of ConA-exposed mice, and IL-15 pre-treatment significantly suppressed their production by NKT cells, with a consequent reduction of eosinophil recruitment [48], which plays a relevant role in the liver damage and necrosis of this experimental model [60]. However, the effect of IL-15 on NKT cells goes beyond the regulation of their cytokine production, since its release from hepatocytes and resident macrophages is essential for NKT cell survival and maintenance in the liver [61,62]. The recent study by Chen et al. suggested an important role of IL-17A in ConA-related liver inflammation since this cytokine was able to induce a strong activation of NKT cells and stimulate their IL-4 production. Notably, the main source of this NKT cell-activating IL-17A was identified in the mucosal-associated invariant T (MAIT) cells residing in the gut and was triggered by a microbial agent [59]. IL-17 can also be expressed by NKT cells (as previously mentioned), and its role as an effector cytokine in liver injury has been described in a murine model of acute experimental hepatitis induced by the administration of αGalCer, characterized by a significant infiltration of neutrophils and proinflammatory monocytes [63].

In addition to underscoring the importance of IL-17 for the specific activation of NKT cells, this aforementioned study by Chen et al. indirectly supported the role of the intestinal microbiota in the pathogenesis of autoimmune hepatitis through the potential contribution of NKT cells [59]. Notably, NKT cells have been recently reported to express receptors binding secondary biliary acids, such as G-protein-coupled bile acid receptor 1 (GPBAR1) [55]. This receptor has been previously described and investigated on macrophages, where its stimulation could impair their production of pro-inflammatory cytokines (such as IL-1, IL-6, and TNF-α) and, in general, promote their polarization towards the anti-inflammatory M2 phenotype [64,65]. In order to explore the role of GPBAR1 in NKT cells, Biagioli et al. investigated ConA-induced hepatitis in the GPBAR1 knock-out mouse, which showed an increased severity of liver injury, supported by a larger expression of inflammatory cytokines (including IFN-γ, TNF-α, and IL-1). They also noticed that such an exacerbation of liver inflammation in this mouse was associated with an increase in the number of NKT cells, which also showed a preferential expression of IFN-γ, to the detriment of the production of IL-10. Moreover, by using a GPBAR1 agonist in ConA-treated wild-type mice, they demonstrated the implication of this receptor in shifting NKT cell functional phenotypes from one producing IFN-γ towards another one with preferential production of IL-10 and, thus, more tolerogenic [56]. Growing evidence supports the implication of gut dysbiosis in the pathogenesis of autoimmune hepatitis [66], and NKT cells (along with other immune cells) may also sense gut dysbiosis through this receptor-binding secondary biliary acids, whose production can be affected by changes in the intestinal microbiota [67].

Finally, it is also worth mentioning the study by Matsumoto et al., who actually adopted a model of murine hepatitis specifically triggered by αGalCer without concomitant ConA administration. In this model, the authors observed the activation of liver NKT cells, of course, which was associated with the concomitant appearance of B-1 lymphocytes responsible for the production of autoantibodies. Here, the authors suggested that NKT cells could directly promote the recruitment and/or activation of B-1 cells, perhaps through the production of IL-4 [68].

**Figure 1:**
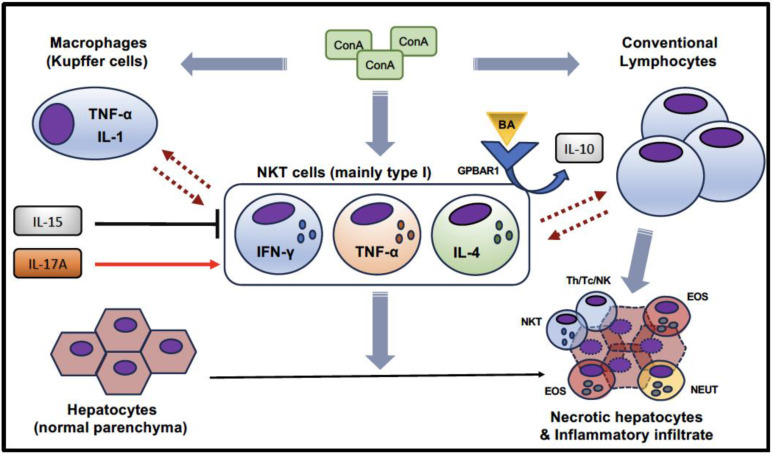
Graphical summary of the contribution of NKT cells in ConA-induced murine hepatitis.

In summary, all these murine experiments support the concept that NKT cells are important, if not critical, players in the pathogenesis of immune-mediated hepatitis. The contribution of NKT cells to ConA-induced hepatitis is graphically summarized in Figure 1. ConA activates resident macrophages (Kupffer cells), conventional T lymphocytes, and NKT cells, which reciprocally interact to create a pro-inflammatory immunological environment [69]. In detail, most studies suggested a relevant role for NKT cells in this pathological setting [43,55], and, conversely, the mitigation of the ConA-induced liver injury was associated with a reduction in NKT cell number and/or activation [44,50,51,53,57,58]. Some studies also provided evidence of their significant cytokine production, especially IFN-γ, TNF-α, and IL-4 [45,46,48,52,56]. Interestingly, Biagioli et al. also showed that the binding of GPBAR1 expressed on NKT cells by secondary biliary acids may alter their cytokine production by reducing the pro-inflammatory cytokines and promoting the expression of IL-10 (which has a tolerogenic effect, probably inducing the emergence of Treg lymphocytes) [56]. Specific studies also suggested the respective inhibitory and stimulatory effects of IL-15 [48] and IL-17 [59] on NKT cell activation. NKT cells contribute to the liver damage in ConA-induced hepatitis by directly targeting hepatocytes but also promoting the recruitment of inflammatory cells, which also include a significant component of eosinophils, due to the production of IL-4 in the hepatic microenvironment [60], in addition to IL-1, IL-6, and TNF-α.

## 3. NKT Cells in Autoimmune Hepatitis: Evidence from Clinical Research

As mentioned in the introduction, there are no pathognomonic features of AIH at the histopathological level, even though typical aspects are represented by intense portal chronic inflammation, and then interface hepatitis, wherein lymphocytic/lympho-plasmocytic infiltrates extend beyond the limiting plate into the adjacent lobule, leading to a severe and progressive loss of hepatocytes and general parenchymal damage [39]. Monocytes/macrophages and conventional T-lymphocytes represent a major component of this inflammatory infiltrate in the liver affected by AIH, wherein B cells can also be demonstrated to a lesser extent [70].

Many research efforts have been dedicated to investigating the properties and/or imbalance of conventional T lymphocytes, especially inside CD4^+^ positive cells, which are considered key players in the immunopathogenesis of AIH since they can include autoreactive lymphocytes and provide cytokines and other inter-cellular signals activating B cells, which are responsible for the production of autoantibodies [40]. Inside this lymphocyte ecosystem of the liver, there are also NKT cells, even though they are relatively less abundant in humans’ liver compared to the murine liver; as previously mentioned, in human beings, type I NKT cells account for <1% of lymphocytes in the liver, and, compared to mice, the hepatic NKT population shows a greater proportion of type II NKT cells [17]. Based on the information from basic research with murine models of autoimmune liver disease, NKT cells are emerging as important players in the immunopathogenesis of this disease, wherein they may also initiate and/or modulate the immune response in addition to serving as effectors of it [71,72]. However, very few studies specifically investigated NKT cells in patients with AIH; indeed, we could retrieve only six human studies providing quantitative and/or qualitative analyses specifically addressed to the NKT cell population in this pathological context, as schematically summarized in Table 2. In total, these studies investigated 216 patients with AIH, including both pediatric and adult cases. From a methodological point of view, they variably analyzed NKT cell homeostasis and cytokine production in these patients [73,74,75,76,77,78].

Chernavsky et al. investigated the expression of Vα24 as an NKT cell marker in the liver tissue and peripheral blood of AIH patients. Here, these cells were respectively increased and reduced compared to controls, suggesting a recruitment of NKT cells in AIH livers, wherein their presence was also detected in the portal spaces and lobular areas by a specific immuno-histochemical staining [73]. A similar (but mRNA expression-based) study approach was used by Solari et al., who also confirmed the increased number of NKT cells in the livers of AIH patients, both at diagnosis and during the therapy-induced disease remission [75]. A reduction of NKT cells circulating in the peripheral blood of AIH patients has also been detected by FACS analysis, especially during active disease, as described in the studies by Ferri et al. and Renand et al. [74,78]. Notably, Ferri et al. also analyzed the cytokine production of NKT cells. However, although there was no difference in the proportion of IFN-γ-producing NKT cells between AIH patients and controls, the former group showed a reduction of NKT cells expressing IL-4, which was more accentuated in active disease [74]. 

Weng et al. published a manuscript aiming to study the type II NKT cells in transgenic mice overexpressing CD1d, which develop spontaneous hepatic chronic inflammation without any additional trigger. They suggested that the hyper-activation of type II NKT cells supported a Th1 polarization of T CD4^+^ cells. Additionally, they included some analysis in AIH patients as an appendix of their research work: here they observed an upregulation of the CD1d expression on the T cell population in AIH patients, which was described in both liver and peripheral blood [76].

Recently, Sebode et al. deeply investigated the phenotype of NKT cells in 74 AIH pediatric and young adult patients. First, they confirmed that in human beings, type II NKT cells are more abundant than type I NKT cells, both in the liver and in the peripheral blood. Whereas no differences were observed in circulating and hepatic type I NKT cells between AIH patients and controls, type II (sulfatide-reactive) NKT cells were significantly increased in both tissues in AIH patients. Moreover, they also reported that type II NKT cells in the liver of AIH patients produced TNF-α more frequently than those from controls, whereas IFN-γ- and IL-4-producing NKT cells were less numerous. Finally, they also showed an elevated expression of CD1d on conventional T cells infiltrating the portal spaces in the liver of AIH patients, which may further promote the activation of these NKT cells [78].

**Figure 2:**
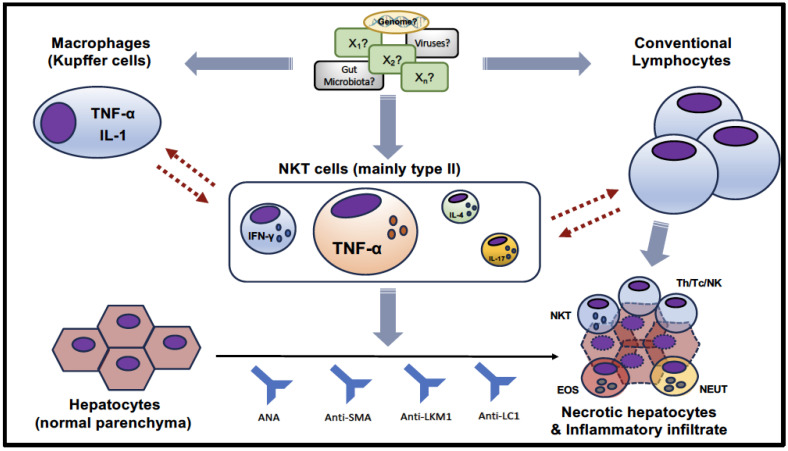
Graphical summary of the available and limited evidence of NKT cell contribution in AIH.

Overall, these studies specifically suggest the involvement of NKT cells in the immunopathogenesis of AIH. The contribution of NKT cells in AIH, based on the few available human studies, is graphically summarized in Figure 2. As a multifactorial disease, several and not completely defined factors (host genes and environmental agents, including a potential contribution of gut dysbiosis and/or viral infections) variably contribute to the immuno-pathogenesis of AIH [3,79]. NKT cells were shown to be numerically increased in the liver of AIH patients, due to their local expansion and, perhaps, recruitment from the bloodstream as well [74,75,76,77]. Here, along with conventional lymphocytes and resident macrophages [79], they participate in the immuno-inflammatory process, even if their exact role has not yet been well defined. Overall, according to the very few available studies that analyzed the cytokine production of NKT cells, these cells showed a remarkable production of TNF-α in AIH patients, whereas the expression of IFN-γ is less evident and that of other cytokines, such as IL-4 and IL-17, could be negligible [74,78]. Indeed, unlike ConA-induced hepatitis, the liver of AIH patients is characterized by an inflammatory infiltrate dominated by lymphocytes and a variable amount of plasma cells, whereas the presence of neutrophils and eosinophils is limited [39,80]. Finally, as mentioned in the introduction, a clear production of specific autoantibodies characterizes patients with AIH [20], even if there is no clear evidence that they are pathogenic [81,82,83].

As said, very few human studies specifically focused their investigations and analyses on NKT cells. Therefore, more methodologically standardized and specific studies are required to further support and clarify the specific role of NKT cells in this human autoimmune disease. Indeed, the evidence emerging from the murine models and, in detail, ConA-induced hepatitis cannot be automatically transferred to AIH due to the obvious immuno-biological differences between species and the specific limitations of the experimental models [84,85], in addition to the clearly different immuno-inflammatory characteristics. Recently, NKT cells have also been investigated in different adult and pediatric autoimmune disorders, and some studies suggested that they could be variably altered in different immune-mediated pathological settings [86,87,88,89].

## 4. Conclusions

Even though the experimental research on murine models suggested a relevant role for NKT cells in immune-mediated hepatic injury, very few studies specifically investigated NKT cell homeostasis in patients with AIH. Such initial and very limited clinical evidence highlighted the presence of several NKT cell alterations in this pathological context, which may even correlate with the disease activity to some extent. However, further studies are definitely needed to investigate the potential role of NKT cell analysis as a disease marker of clinical relevance and, if so, the immunological and molecular mechanisms by which NKT cells contribute to the pathogenesis of autoimmune hepatitis, also with respect to different epidemiological and clinical aspects (e.g., AIH type 1 vs. type 2, adults vs. children).

## Figures and Tables

**Table 1:** Main studies assessing the NKT cell number and/or activation in the murine model of Concanavalin-A hepatitis.

Authors, Year, Country	Transgenic Mice	Mouse Background	AIH Model	Analytical Methods (NKT Cells)	NKT Cell Identification	Aim	NKT-Related Findings	Additional Findings	Conclusion
**Toyabe** * et al., **1997**, **Japan** [42]	n/a	n/a	ConA	n/a	n/a	To assess the role of lymphocyte subsets and specific inflammatory cytokines.	n/a	n/a	“Although CD4+ cells in the liver and their production of TNF-alpha are the direct effectors of Con A-induced hepatic injury, liver NK1+ T cells also play an important role in this hepatitis model.”
**Kaneko** et al., **2000**, **Japan** [43]	gld/gld Vα14^−/−^ RAG^−/−^ IL-4^−/−^ IFNγ^−/−^ Perforin^−/−^	C57BL/6	ConA	FACS	TCRβ^+^ NK1.1^+^	To investigate the relevance of NKT cells in the disease development.	Vα14 NKT cells are required for the development of hepatitis and can do it in absence of conventional T cells, B cells, and NK cells.	Vα14 NKT cells from either Fas^−/−^ or gld/gld mutant mice cannot induce hepatitis. IL-4 produced by NKT can upregulate FasL and granzyme B expression on these same cells in an autocrine fashion, which are important for the induction of the hepatitis.	NKT cells and autocrine production of IL-4 are required and sufficient for inducing ConA–induced hepatitis.
**Takeda** et al., **2000** **Japan** [44]	gld/gld Perforin^−/−^ CD1d^−/−^	C57BL/6	ConA	FACS	CD3^+^ NK1.1^+^	To investigate the role of liver NKT cells in the disease development.	Hepatitis was markedly reduced in CD1d^−/−^ mice compared to wild type mice. Adoptive transfer of wild-type NKT cells (but not from gld/gld mice) can induce hepatitis in CD1d^−/−^ mice.	The expression of functional FasL on hepatic NKT cells plays a critical role in hepatitis induction.	NKT cells have a critical role in the induction of ConA hepatitis, and functional FasL expression on NKT cells is essential to cause liver injury.
**Yamanaka** et al., **2004**, **Japan** [45]	WSX-1^−/−^	C57BL/6	ConA	FACS	CD3^+^ NK1.1^+^	To investigate the role of WSX-1 in NKT cells.	WSX-1-deficient NKT cells can produce more IFN-γ and IL-4 than in wild-type NKT both in vitro and in vivo.	WSX-1-deficient mice with hepatitis showed higher production of several inflammatory cytokines, including IL-1, IL-6, and TNF-α.	WSX-1 does not affect the NKT development but has an inhibitory effect on their IFN-γ and IL-4 production.
**Biburger** et al., **2005**, **Germany** [46]	—	C57BL/6 BALB/c	ConA (+αGalCer)	FACS	CD3^+^ NK1.1^+^	To investigate and compare the cytokine production in αGalCer- and ConA-induced liver injury.	In αGalCer-induced hepatitis (unlike ConA-induced hepatitis) TNF-α is produced by intrahepatic lymphocytes, especially NKT cells.	Compared to BALB/c strain, C57BL/6 mice are more sensitive to αGalCer-induced liver injury and showed higher TNF-α production and FasL expression on NKT cells.	Unlike in ConA-induced hepatitis, in αGalCer-mediated hepatitis NKT cells can produce TNF-α, which is required to induce liver injury.
**Kawamura** et al., **2006**, **USA** [47]	P2X7R^−/−^ ART-2^−/−^	C57BL/6	ConA	FACS	CD3^+^ NK1.1^+^	To investigate the effects of NAD on NKT cells.	NAD has an inhibitory action on “naïve” NKT cells via stimulation of P2X7 receptors. However, an opposite effect can be observed on αGalCer-primed NKT cells in the context of ConA-induced hepatitis.	Mice knockout for P2X7 receptors have a reduced liver injury in the experimental model, as well as those lacking ADP-ribosyl-transferase.	Purinergic receptors can regulate NKT cells activation in autoimmune hepatitis.
**Li** et al., **2006**, **China** [48]	CD1d^−/−^	C57BL/6	ConA	FACS	NK1.1^+^ CD3^+^	To assess the role of IL-15 and KKT cells	the depletion of NKT cells impairs liver injury, which can be restored by adoptive transfer of purified NKT cells. the transfer of NKT cells from wild-type to CD1d^−/−^ mice, induces liver injury.	IL-15 pretreatment can significantly inhibit the NKT Cell-related production of IL-4, IL-5, and TNF-α.	IL-15 has a protective effect in Con A–induced liver injury through an inhibitory effect on the production of IL-4, IL-5, and TNF-α by NKT cells.
**Mencarelli** et al., **2009**, **Italy** [49]	FXR^−/−^	C57BL/6	ConA	FACS	NK1.1^+^ CD3^+^	To assess the role of the Bile Acid Sensor Farnesoid X Receptor	FXR is expressed by NKT cells, and its absence favors the production of ostepontin by these cells.	FXR^−/−^ mouse shows enhanced susceptibility to ConA-mediated hepatitis, in addition to being prone to spontaneous liver disease.	Bile acid sensor FXR can modulate the activation of liver NKT cells and their osteopontin production.
**Fang** et al., **2012**, **USA** [50]	PKC-θ^−/−^	C57BL/6	ConA	FACS	CD3^+^ CD1d/ tetramer^+^	To assess the role of PKC-θ in NKT cell-mediated liver injury.	NKT cell development is defective in PKC-θ^−/−^ mice. PKC-θ is required for thymic NKT cell development.	PKC-θ^−/−^ mice show lower levels of inflammatory cytokines.	Deletion of PKC-θ likely impairs liver injury, due to the developmental defect of NKT cells
**Jung** et al., **2012**, **Republic of** **Korea** [51]	VSIG4^−/−^	C57BL/6	ConA	FACS	TCRβ^+^ NK1.1^+^	To investigate the role of VSIG4 cells in liver disease	In absence of VSIG4, liver T cells and NKT cells are more responsive to antigen-specific stimulation with tolerance impairment.	VSIG4 expression by Kupffer cells inhibits in vitro the production of cytokines from NKT cells (including IL-4, TNF-α, IFN-γ).	VSIG41 expressed by Kupffer cells is implicated in inducing and maintaining the tolerance of liver T and NKT cells
**Wang** et al., **2014**, **China** [52]	Stat4^−/−^ Il12b^−/−^ Il12a^−/−^	C57BL/6	ConA	FACS	CD3^+^ NK1.1 CD1d/ tetramer^+^	To investigate STAT4 activation in this pathological context.	NKT Cells from Stat4^−/−^ mice showed higher expression of FasL and greater cytotoxicity against hepatocytes compared to wild-type mice.	STAT4-related IL-12 activation inhibits the expression of FasL on NKT cells.	IL-12 activation via STAT4 in NKT cells (in addition to that in T cells and macrophages) can modulate and ameliorate the hepatic injury.
**Filliol** et al., **2017**, **France** [53]	Parp1^−/−^ Parp2^−/−^	C57BL/6	ConA	FACS	CD3^+^ TCRVβ^+^ NK1.1^+^ CD1d/ tetramer^+^	To define the roles of PARP1 and PARP2 proteins.	Parp2^−/−^ mice showed a significant reduction of liver NKT cells.	PARP2, but not PARP1, deficiency protects mice from liver injury	The systemic reduction of NKT cells in Parp^−/−^ mice attenuates the liver injury.
**Hines** et al., **2018**, **USA** [54]	PPARα^−/−^	C57BL/6	ConA	FACS	TCRβ^+^ NK1.1^+^	To examine PPARα in the pathogenesis of liver injury.	PPARα is implicated in the recruitment and/or survival of NKT cells: its deficiency is associated to a resistance liver injury along with a reduction of NKT cells.	PPARα deficiency is associated to a reduction of expression of IL15 (not CD1d) in the liver. Moreover, the production of cytokines, especially IFN-γ, in PPARα^−/−^ mice upon αGalCer administration was impaired.	The protective effect of PPARα deficiency is mainly mediated by the impairment of NKT cell number and their activation.
**Tabet** et al., **2018**, **France** [55]	—	C57BL/6	ConA	FACS	CD3^+^ TCRVβ^+^ NK1.1^+^	To investigate the impact of chlordecone on the progression of liver injury in this experimental model.	Chlordecone significantly amplified the liver damage, which was also associated to an increase of liver NKT cells, without any other remarkable numerical change in other immune cells, including neutrophils, macrophages, and CD4, CD8, NK, B lymphocytes.	—	NKT cells plays a relevant role in the entity of liver injury in this pathological context.
**Biagioli** et al., **2019**, **Italy** [56]	GPBAR1^−/−^ IL-10^−/−^	C57BL/6	ConA	Microbeads purification	NK1.1^+^ CD3^+^ CD1d/ tetramer^+^	To explore the role of GPBAR1 in the regulation of liver NKT cells.	The absence of GPBAR1 worsened liver injury and was associated with an NKT phenotype polarization towards type I NKT cells with production of IFN-γ.	NKT cells from GPBAR1^−/−^ mice resulted to be sufficient for causing liver injury when administered to wild-type mice. GPBAR1 agonists can reduce the liver injury and redirect NKT cells toward a regulatory phenotype, producing IL-10.	The NKT cell immune-phenotype and their cytokine profile can modulate the liver injury in this pathological context.
**Gao** et al., **2019** **China** [57]	—	C57BL/6	ConA	FACS	CD3^+^ NK1.1^+^	To examine the mechanisms of DG protection in this experimental model.	DG pretreatment significantly reduced the number of NKT cells in the liver and, conversely, increased the number of hepatic Tregs.	—	NKT cells reduction is one of the mechanisms by which DG can reduce liver injury in this pathological model.
**Gao** et al., **2020**, **China** [58]	CD1d^−/−^ LXRα^−/−^ LXRα KI (FABP)-VP-LXRα^−/−^	C57BL/6	ConA	FACS	CD3^+^ NK1.1^+^	To study the role of LXR in the pathogenesis of liver injury.	Hepatic NKT cells are necessary and sufficient to sensitize LXRα-KI mice to ConA-induced hepatitis.	The sensitizing effect of LXRα activation depends on NKT cell activation and production of IFN-γ.	LXRα plays an important role in this pathological context, and NKT cells and their cytokine production have a pathogenic relevance.
**Chen** et al., **2011**, **China** [59]	IL-17a/ EGfp KI IL-17a^−/−^	C57BL/6	ConA	FACS	NK1.1^+^ TCβ^+^	To assess the role of IL-17A in this hepatitis model upon exposure to Salmonella typhimurium in the gut.	NKT cells were hyper-activated by IL-17A in the liver of mice with hepatitis and exposed to S. typhimurium administration group.	Intra-cellular IFN-γ and IL-4 were increased in NKT cells of these mice, as well as the expression of FasL.	MAIT-produced IL-17 can worsen liver injury by mainly activating NKT cells.

**Abbreviations:** AIH—autoimmune hepatitis; ConA—Concanavalin A; CD4—cluster of differentiation 4; ^−/−^—knock-out; KI—knock-in; TNF-α—tumor necrosis factor α; NK1+ T—natural killer 1+ T cells; gld—generalized lymphoproliferative disease; Vα14 NKT—natural killer T cells expressing an invariant T-cell receptor; RAG—recombination activating gene; IL-4—interleukin 4; IFNγ—interferon γ; C57BL/6—inbred strain; FACS—fluorescence-activated cell sorting; TCRβ+—T-cell receptor β chain; FasL—Fas ligand; NK—natural killer cells; CD1d—cluster of differentiation 1d; CD3—cluster of differentiation 3; WSX-1 knockout—MRL/*lpr* mice deficient for IL-27 receptor; IL-1—interleukin 1; IL-6—interleukin 6; BALB/c—inbred strain; α-GalCer—α-Galactosylceramide; P2X7R—purinergic receptor; ART-2—ADP-ribosyltransferase 2; NAD—nicotinamide adenine dinucleotide; IL-15—interleukin 15; IL-5—interleukin 5; FXR—farnesoid X receptor; PKC-θ—protein kinase C θ; VSIG4—V-set and Ig domain-containing 4 protein; STAT—signal transducer and activator of transcription protein; IL-12—interleukin 12; PARP—poly (ADP-ribose) polymerase; PPARα—peroxisome proliferator activated receptor α; GPBAR1—G-protein-coupled bile acid receptor 1; IL-10—interleukin 10; DG—diammonium glycyrrhizinate; LXRα—liver X receptor α; FABP-VP-LXRα—fatty acid binding protein knock-in transgenic mice; IL-17A—interleukin 17A; IL-17a/EGFP—interleukin 17A/enchanced green fluorescent protein mice. Notes: *—The full article of this study could not be retrieved.

**Table 2:** Clinical studies describing NKT cell homeostasis and/or activation in AIH patients.

Authors, Year, Country	Study Design	Study Population (N)	AIH (Gender & Age)	Subgroup (1)	Subgroup (2)	Controls [N; Gender; Age]	NKT Cells Analysis [Tissue]	Results	Conclusion
**Chernavsky** et al., **2004**, **Argentina** [73]	Cross- sectional	PAH (**N** = 25)	**M:F** = 10:15 ***Median*** ***(range)*** 10 yrs. (6–15)	—	—	*Children with HCV chronic hepatitis* **N** = 6 **M:F** = n/a **Age**: n/a *Cadaveric* *donors* **N** = 9 **M:F** = 10:15 ***Median (range)*** 16 yrs. (7–30)	Vα24 mRNA expression analysis [PBMC, liver] Vα24 immuno- histochemical-staining [liver]	- Vα24 expression is upregulated in the liver of PAH patients in comparison with control livers from cadaveric donors (2.46 ± 0.44 vs. 0.14 ± 0.07, *p* = 0.0007). - Vα24 expression was reduced in PBMC of PAH patients compared to control children (0.73 ± 0.16 vs. 2.13 ± 0.65, *p* = 0.041). - The immunostaining confirmed the presence of Vα24+ cells in the lobular area and in the portal spaces, whereas in cadaveric controls and control children these cells were restricted to the lobular areas.	NKT cells presence is increased in PAH liver and their concomitant decrease in the circulation could reflect the liver recruitment. Along with the immunostaining results, these data suggest that NKT cells can have a role in PAH liver injury.
**Ferri** et al., **2010**, **Italy** [74]	Cross- sectional	AIH-1 (**N** = 47)	**M:F** = 10:37 ***Median*** ***(range)*** 48 yrs. (17–79)	*Active patients* **N** = 16	*Remission* *patients* **N** = 31	*“Healthy* *controls*” **N** = 28 **M:F** = 9:19 ***Median (range)*** 39 yrs. (23–58)	CD3^+^ CD56^+^ Cells FACS analysis [PBMC]	- NKT cells were significantly reduced in AIH patients compared to controls (8.26 ± 1.09 vs. 16.19 ± 1.83; *p* < 0.005). - NKT cells were significantly reduced in both active and remission patients AIH compared to controls (respectively, 6.16 ± 1.17 and 9.32 ± 1.50, vs. 16.19 ± 1.83; *p* = 0.001 and *p* = 0.005). - The stimulation of PBMC with αGalCer resulted in a greater expansion of CD3+CD56+ cells in AIH patients than in controls (567 ± 153% vs. 190 ± 25%). - Whereas there was no difference in the NKT intra-cellular content of IFN-γ between AIH patients and controls, that of IL-4 resulted to lower in AIH patients compared to controls. The number of granzyme B+ NKT cells was increased in AIH patients compared to the controls (51.20 ± 4.31 vs. 37.52 ± 4.19; *p* < 0.05).	The number of NKT cells is significantly reduced in active patients and is only partially restored during the disease remission. Circulating NKT cells from AIH patients were reported to produce lower amounts of IL-4, but are more responsive to the antigenic stimulation. Overall, NKT cells from AIH patients show altered homeostasis and responses.
**Solari** et al., **2010**, **Argentina** [75]	Cross- sectional	AIH-1 (**N** = 34)	See subgroups	*Active patients* **N** = 12 **M:F** = 2:10 ***Median*** ***(range)*** 7.7. yrs. (3–16)	*Remission* *patients* **N** = 22 **M:F** = 6:16 ***Median*** ***(range)*** 12.8. yrs. (7–20)	*Cadaveric* *donors* **N** = 14 **M:F** = n/a ***Median*** ***(range)*** 16 yrs. (7–30)	Vα24 mRNA expression analysis [liver]	- Vα24 mRNA expression was significantly increased in both active (*p* = 0.002) and inactive (*p* = 0.03) AIH groups compared to controls. No significant differences between active and inactive AIH patients were observed.	An increased NKT presence in the liver has been observed in AIH patients, which suggests their involvement in the inflammatory response in AIH-1 patients.
**Weng** et al., **2017**, **USA** [76] *	Cross- sectional	AIH (**N** = 11)	**M:F** = 0:11 ***Mean*** 51.6 yrs. (38–60)	—	—	“Healthy donors” **N** = 10 **M:F** = n/a **Age**: n/a	CD1d Immuno- Histochemical-staining [liver] FACS? [PBMC]	- Circulating T cells from AIH patients showed higher CD69 expression than control, which in turn displayed higher CD1d expression. - In livers from AIH patients, a proportion of infiltrating T cells expressed high level of CD1d.	These data could suggest an upregulation of CD1d on T cells and, thus, an increased recruitment and activation of NKT cells in AIH patients.
**Renand** et al., **2018**, **France** [77]	Retrospective (biobank)	AIH (**N** = 25)	See subgroups	*Active patients* **N** = 14 **M:F** = 6:8 ***Mean*** ***(range)*** 62 yrs. (17–74)	*Remission* *patients* **N** = 11 **M:F** = 2:9 ***Mean*** ***(range)*** 57 yrs. (43–79)	*“Healthy donors”* **N** = 14 **M:F** = 6:8 ***Mean*** ***(range)*** 49 yrs. (22–65)	TCR V*α*24J*α*18 FACS analysis [PBMC]	- Circulating NKT cells were significantly reduced in new-onset (active) AIH patients compared to healthy donors (*p* < 0.001). - The frequency of NKT cells was not restored in inactive AIH patients during immunosuppressive therapy.	The altered homeostasis of NKT cells could suggest a role of these cell population in the immune dysregulation observed in AIH patients.
**Sebode** et al., **2019**, **Germany** [78]	Cross- sectional	AIH (**N** = 74)	**N** = 46 [PBMC] **M:F** = 11:35 ***Median*** ***(range)*** 47 yrs. (22–77) **N** = 28 [liver] **M:F** = 8:20 ***Median*** ***(range)*** 53 yrs. (24–78)	*Active patients* **N** = 13 [PBMC] **N** = 16 [liver]	*Remission patients* **N** = 38 [PBMC] **N** = 12 [liver]	*DILI* **N** = 10 [PBMC] **N** = 11 [liver] *“Healthy donors”* **N** = 39 [PBMC] **N** = 17 [liver]	αGalCer-loaded tetramer and sulfatide-loaded tetramers FACS analysis [PBMC, liver] CD1d immuno- histochemical-staining [liver]	- The frequency of type II NKT cells in blood was significantly increased in AIH patients compared to controls and DILI patients (0.11% vs. 0.05% and 0.06%; *p* < 0.01) - Liver type II NKT cells were significantly more frequent in AIH patients than in healthy donors and DILI patients (3.78% vs. 1.90% and 2.03%; *p* < 0.05). - There was no significant difference in circulating or liver type II NKT cell numbers between active and inactive AIH patients. - The number of circulating and liver type I NKT cells was very low and did not differ significantly among patient groups. - Circulating type II NKT cells in AIH patients produced mainly TNF-α and, to a lesser extent, IFN-γ, IL-17, and IL-4. - In AIH patients, liver type II NKT cells showed a different cytokine profile compared to healthy controls: TNF-α and IFN-γ production were respectively higher and lower (*p* < 0.05). The expression of IL-4 was basically absent, whereas it was present in healthy subjects, even if it was low (*p* < 0.05). IL-17 expression in type II NKT was not different between these groups.	Type II NKT cells in livers of AIH patients showed a proinflammatory cytokine profile. Moreover, infiltrating T cells observed in the portal space of AIH patients’ livers resulted to overexpress CD1d. Therefore, type II NKT cells are supposed to promote the inflammation in patient with AIH.

**Abbreviations:** yrs.—years; PAH—pediatric autoimmune hepatitis; M—males; F—females; HCV—hepatitis C virus; PBMC—peripheral blood mononuclear cells; NKT—Natural Killer T cells; AIH-1—type I autoimmune hepatitis; αGalCer—α-galactosylceramide; IFN-γ—interferon γ; IL-4—interleukin 4; TCR V*α*24J*α*18—invariant V*α*24J*α*18 T cell receptor; DILI—drug-induced liver injury; TNF-α—tumor necrosis factor α; IL-17—interleukin 17. Notes: *—This article mainly described murine experiments, but also included a minor part describing results from AIH patients; ?—the methods related to human sample analysis are not fully described, and the precise reagents used for FACS analysis could not be retrieved from the paper.

## Data Availability

Not applicable.
